# Modeling Compound Impression Taking*Read before the Academy of Stomatology, Philadelphia. Nov., 1920. Reprinted in *Cosmos*, June, 1921.

**Published:** 1921-10

**Authors:** Edward Kennedy

**Affiliations:** New York


### MODELING COMPOUND IMPRESSION TAKING*

BY EDWARD KENNEDY, D.D.S., NEW YORK

The subject I have chosen to present is too large to do justice to in one evening, so I shall be compelled to give my reasons for the use of modeling compound and touch only lightly on the technique. I claim no originality for the technique, with the exception of a few points in the partial impression work; but I would like to make mention of the names of Drs. Tench and Giffen and Mr. Supplee, who have done so much in developing practical methods along the lines of Dr. Greene's teaching.

After taking impressions in plaster for ten years, I changed to modeling compound because in many of the cases in which I failed in plaster, while reconstructing them, I had much better results when I used modeling compound.

As a further proof of its advantages, take a denture which is correct in appearance and occlusion but lacks the proper suction and line it with modeling compound, and in almost every instance you will find the patient can get along comfortably, showing that, other things being equal, the impression taken in modeling compound will give the better result.

Of course this brings in the matter of personal equation and arguments to be made pro and con on that factor alone. It is the opinion of the writer that we will not get very far with dentistry if we adhere to this method of treating the subject; that is, accepting a method because some one man

* Read before the Academy of Stomatology, Philadelphia, Nov., 1920. Reprinted in *Cosmos*, June, 1921.

testifies to its success in his hands. We must develop standardized technique, with standardized materials and try them out under actual practice conditions, having a number of different men making the tests and accepting that method to teach which gives the best results to the group. This can only be done by following Dr. Giffen's suggestion and forming clinic clubs for scientific study and not for the purpose of turning them into social or political organizations or to the personal aggrandizement of one man who has some method to expound or some instruments to sell.

STANDARDIZATION

We must first standardize our nomenclature. Dr. Greene in writing his book made the mistake of using terms which were understood by no one but himself.

We must classify our cases if we hope to have some definite method of procedure. The subject of orthodontia made little progress until Angle came forth with his classification. It is some satisfaction to know that the work which Mr. Supplee started in classifying prosthetic cases has been carried farther by Dr. House and others in a very complete manner. I know of no one who has classified partial cases, but hope that this will also soon be done. And lastly, we must have a standardization of materials so that each dentist trying a suggested method will be able to duplicate the conditions laid down in that method and then we will know just how much the personal equation enters into a result.

Modeling compound being a standardized material is the greatest reason for its use in impressions. In almost every instance where a man fails in using plaster, he blames it on the material rather than upon his skill, and I believe that the element of guess is too great to make it a reliable material. Each mix of plaster varies from the preceding one and this is due to many factors, some of which are not within the control of the dentist. The quality of the gypsum rock varies in each mine; the degree to which it is calcined causes a variation in the setting; the process of sifting it varies with each individual operator and even the plaster at the top of the barrel sets differently from that at the bottom, due to the fact that the coarser granules separate from the finer in the barrel. The temperature of the air, the amount of moisture in the air, the amount and temperature of the water used in the mixing of the plaster are all factors which cause variations and the dentist is only able to control a few of these. The expansion and contraction of the plaster tend to alter the shape of the impression in accordance with the length of time the cast is poured after the impression is taken. We have no accurate data on this subject, but I have a slide with me which illustrates just what happens, and I know of no way of accurately overcoming it.

### REQUISITES OF AN IDEAL IMPRESSION MATERIAL

An ideal impression material must be one that is capable of reproducing the finer lines of the mouth. Plaster does this, but with no greater degree of accuracy than does the modeling compound. It must be a material capable of reproducing the undercuts in a case when it is wanted and a material in which we can eradicate them when so desired without the chance of destroying the accuracy of the other portion of the impression.

### COMPARISON OF PLASTER AND MODELING COMPOUND

Plaster does not answer this requirement. Modeling compound does. It must be a material in which we can control the setting qualities. Plaster does not answer this requirement while modeling compound does. Modeling compound can be heated to a definite degree, say 160 degrees F., which is the best degree of heat for impression taking, and can be chilled with a spray bottle or ice water while in the mouth to a stone hardness and then it can be removed without distortion. It must be a material which has a pleasant flavor and does not nauseate the patient.

Plaster is very objectionable to most patients and the fear of choking in timid patients is always present. I have had a number of patients say to me after I have finished taking impressions in modeling compound: "When are you going to use that horrid plaster?"

But the greatest reason for using modeling compound is the fact that we can correct the impression for any inaccuracies that may occur or, what is more important, equalize the pressure on the hard and soft areas of the mouth producing a plate that has nearly a perfect suction. I have seen a number of lower plates that have so great a suction that a twisting force had to be used to get them out of the mouth.

The fitting of the plates after vulcanization is practically eliminated and this is particularly noticeable in partial impression work. I have seen men take anywhere from one-half to an hour and a half in fitting their partial plates and then not be sure that they had not weakened the plates to such a degree that they would not stand up under the force of mastication.

These same men will condemn the use of modeling compound on the strength of the fact that it takes longer to take an impression in modeling compound than it does in plaster. While I will acknowledge that some modeling compound impressions may take half an hour to obtain, yet the advocates of plaster will totally disregard the time spent in fitting the pieces of their broken impression together and likewise the time spent in fitting the plate to the mouth.

I believe it takes more time to learn how to use modeling compound than it does to learn how to use plaster, but after a dentist has mastered the use of compound he approaches his patients with a certainty of success, which breeds confidence and this is one-half the battle of plate work.

After the impression is taken, the dentist does not have to wait until the plate is finished to know whether or not the impression is accurate, but can test it again and again and correct any weak points without destroying the good ones.

Before taking up this work he must forget his technique of plaster impressions and try not to apply any step which he has learned in plaster to modeling compound or he will have an absolute failure. I shall briefly outline the method of taking a full upper impression and then a full lower and show the different steps by means of lantern slides.

### TECHNIQUE OF TAKING A FULL UPPER IMPRESSION

When a patient presents, it is my custom to take study impressions. Casts made from these impressions will help us select a tray so that we can obtain one that is more accurately fitting than when we just guess. Also, any irregularities in the mouth may be noted and in many instances the patient's fear of the impression will be dispelled, as we do not need to take an impression which will cover as much area as the final plate. It permits us to judge our patients and if we feel that the case or the patient will present difficulties we can allow for them in our estimates.

On the study case a tray is swaged, which can be quickly and accurately done by means of one of the swagers that are on the market: the Ash, the Olivian or a shot swager; or the tray can be burnished to the cast with a large burnisher. The tray should be made of Ash & Sons tray metal or 22-gage aluminum.

About half a sheet of modeling compound is heated in water to about 150 degrees F., placed in the tray and shaped approximately to fit the study cast, which has been wet with cold water. Or an approximate adaptation can be made by means of the fingers. The impression is then placed in the heater (I use a Monson heater for controlling the temperature) and when the surface is softened enough it is forced upward and forward in the mouth with a wave-like motion. The patient is instructed to swallow so as to turn the compound down at the posterior edge and when it is slightly hardened the finger is placed between the lip and the compound, forcing the compound upward against the muscle attachments; then the patient is instructed to go through any normal muscular movement that might be used in masticating or speaking.

The impression is then chilled and carefully dislodged, placed in cold water and any surplus material that extends beyond the plate outline is trimmed off. This mostly occurs at the posterior edge and one can see the muscles of the plate begin to turn the impression downward. This is the point where the plate should end. If the impression is thicker on the rims than it is the intention to make the finished denture, these also should be trimmed to the correct thickness. If we intend to build plumpers on the plate they should be added at this time.

In other words, the finished impression should cover the same area and be practically the same size as the finished denture. The impression is heated over the Bunsen burner wherever we have cut it with the knife, dipped in the hot water and the patient instructed to again go through the necessary movements of speaking, swallowing, singing; in fact, any movement that the patient is apt to make with the finished denture. If the impression falls it must be corrected at this stage, and we must determine where there is an air leakage and add more material at this point, or if there are undue pressures on the hard spots causing the plate to rock, they must be relieved by scraping or additions to the soft areas. In a great majority of cases it will be necessary to make slight additions to the heels or on the edges.

I have adopted Dr. Tench's idea of using for this purpose the black wax upon which the teeth come to us from the supply house. This is melted on the spots which need slight additions, lightly passed through the flame of a Bunsen burner, so lightly as not to soften the compound underneath, and then placed in the mouth for a final trial.

If it tests correctly, carefully remove and place in cold water until ready to pour.

### TECHNIQUE OF TAKING A FULL LOWER IMPRESSION

The lower impression is slightly more difficult, but is made in a similar manner. First a study cast is made and tray fitted to it. The compound is placed in the tray and either fitted approximately with the fingers to the cast, or the cast is wet in cold water and the hot compound in the tray shaped over the cast. It is replaced in the heater until it is heated throughout and it is then placed in the patient's mouth, getting an impression without having the patient go through any muscular movements. It is then chilled with ice water or with a spray, lifted out of the water and trimmed approximately to the lines that the plate will cover. If it does not show suction it is corrected according to the diagram. The areas over the heel are corrected first; next about the frenum under the tongue; next the lip; next the areas between the heels and the frenum, and lastly the areas where the attachments of the muscles of the cheeks come. I find that the tongue readily trims the impression, but the areas of the lip and the buccal muscle will have to be assisted by the fingers.

In most cases, it will be impossible to extend the impression over the mylohyoid ridge, as the mouth becomes very sore at this point if this is done. If very flat ridges are present and the operator feels he needs extra hold, he can sometimes extend the plate directly backward from the mylohyoid ridge, but not downward. When the impression tests correctly it is taken out and chilled in ice water and the cast made. Sometimes after working with impressions, a slight rock develops. In most instances it is best to begin over again, but there are two ways of correcting it. One by taking a syringeful of hot water and squirting on the internal surface of the impression so that just the surface is warmed, but not the body. It is then placed in the mouth and pressure is brought to bear evenly on both sides of it. Another way is to dry your impression, take a thin sheet of compound, heat and place in the impression. A Bunsen flame is lightly passed over it so that it melts evenly over the surface of the impression. It is then placed in the heater for a second to equalize the temperature, and replaced in the mouth with a steady downward pressure on both sides over the area of the premolar regions. This process is quite similar to that of rebasing the plate. A number of impressions, which would have otherwise been spoiled, have been saved by this method.

Sometimes we notice an escape of air at a point on the impression which will be shown by small air bubbles escaping when pressure is applied. This can be corrected by applying the black wax at that point without danger of changing the work which has already been done. The great fundamental principle to be observed in this work is to get an impression of the hard areas with no rock of the impression. When this has been obtained, the impression must now be extended just onto the softer areas, but not onto the movable areas. This gives a valve fit at the edges and prevents the leakage of air.

The steps in the impression work are most beautifully illustrated in the books which Drs. Clapp and Tench have written, and I would advise anyone who intends to take up the work to study these books carefully. Also, there are many helpful suggestions in Dr. Greene's book, but the nomenclature rather clouds the descriptive text.

I would suggest to beginners who are skeptical as to the merits of modeling compound to select one of the plates they have made which is not working successfully, trim the ridges down so that the muscles will not dislodge the plate, place a thin sheet of modeling compound in the inside of the plate (sheets come already prepared for this purpose), build up the edges with a tracing stick and place in the heater until the compound becomes heated evenly throughout. The heater must be rather hot; not less than 150 degrees F. Then place in the mouth and have the patient close upon the teeth, making any motion he desires with the lips and cheeks, still holding the plate tightly in place with the lower teeth. Also have him swallow, and when thoroughly chilled lift the plate out of the mouth and trim the compound. In other words, use the old plate as an impression tray. This can be flasked in the usual manner of relining plates, and rubber substituted for the compound, vulcanized and finished. In every instance where I have used this method, I have been able to better the fit of the plate and in many instances after finishing new plates I have found that if they lack a proper degree of suction, or the suction breaks under the strain of mastication, it can always be improved.

We use the natural color rubber in making the base, as it is better tolerated by the tissues of the mouth, being devoid of coloring matter. Also if you reline a plate it looks less "patchy" than any color but black.

